# Chemoautotrophic Carbon Fixation Rates and Active Bacterial Communities in Intertidal Marine Sediments

**DOI:** 10.1371/journal.pone.0101443

**Published:** 2014-07-08

**Authors:** Henricus T. S. Boschker, Diana Vasquez-Cardenas, Henk Bolhuis, Tanja W. C. Moerdijk-Poortvliet, Leon Moodley

**Affiliations:** 1 Department of Marine Microbiology, Royal Netherlands Institute for Sea Research (NIOZ), Yerseke, The Netherlands; 2 Marine Environment Group, International Research Institute of Stavanger (IRIS), Randaberg, Norway; Universidade Federal do Rio de Janeiro, Brazil

## Abstract

Chemoautotrophy has been little studied in typical coastal marine sediments, but may be an important component of carbon recycling as intense anaerobic mineralization processes in these sediments lead to accumulation of high amounts of reduced compounds, such as sulfides and ammonium. We studied chemoautotrophy by measuring dark-fixation of ^13^C-bicarbonate into phospholipid derived fatty acid (PLFA) biomarkers at two coastal sediment sites with contrasting sulfur chemistry in the Eastern Scheldt estuary, the Netherlands. At one site where free sulfide accumulated in the pore water right to the top of the sediment, PLFA labeling was restricted to compounds typically found in sulfur and ammonium oxidizing bacteria. At the other site, with no detectable free sulfide in the pore water, a very different PLFA labeling pattern was found with high amounts of label in branched i- and a-PLFA besides the typical compounds for sulfur and ammonium oxidizing bacteria. This suggests that other types of chemoautotrophic bacteria were also active, most likely Deltaproteobacteria related to sulfate reducers. Maximum rates of chemoautotrophy were detected in first 1 to 2 centimeters of both sediments and chemosynthetic biomass production was high ranging from 3 to 36 mmol C m^−2^ d^−1^. Average dark carbon fixation to sediment oxygen uptake ratios were 0.22±0.07 mol C (mol O_2_)^−1^, which is in the range of the maximum growth yields reported for sulfur oxidizing bacteria indicating highly efficient growth. Chemoautotrophic biomass production was similar to carbon mineralization rates in the top of the free sulfide site, suggesting that chemoautotrophic bacteria could play a crucial role in the microbial food web and labeling in eukaryotic poly-unsaturated PLFA was indeed detectable. Our study shows that dark carbon fixation by chemoautotrophic bacteria is a major process in the carbon cycle of coastal sediments, and should therefore receive more attention in future studies on sediment biogeochemistry and microbial ecology.

## Introduction

Reoxidation of reduced intermediates like sulfide and ammonium formed during anaerobic mineralization processes is an important process in coastal marine sediments. Oxygen is typically only found in the top millimeters of these sediments and along macrofauna burrows [1], and carbon mineralization proceeds in general by anaerobic processes primarily sulfate reduction. This results in the production and accumulation of large amounts of reduced compounds such as various forms of reduced sulfur and ammonium [2]. In typical coastal sediments, free sulfide in the porewater is however often only detected below a couple of centimeters as it quickly reacts with iron hydroxides forming iron sulfide (FeS) or pyrite (FeS_2_) [3]. Only in very active sediments or sediments containing little reactive iron, free sulfide can be found near the oxic top layer [3]. Long term burial of reduced compounds is thought to be a minor process [3] and they are mostly transported to more oxidized horizons by either diffusion or bioturbation [4]. Oxygen is eventually the main oxidant of these reduced compounds although intermediate reoxidation steps by a variety of anaerobic pathways using nitrate or iron and manganese oxides may also be important [3]. It is estimated that reoxidation processes on average explain 70% of the sediment oxygen flux in shelf sediments [5] and this value is expected to be higher in active intertidal areas as anaerobic mineralization will be more important.

Many of the known prokaryotes involved in reoxidation processes are chemo(litho)autotrophs that use the energy gained from inorganic reactions to grow by fixing inorganic carbon in the dark [6]. Chemoautotrophic carbon fixation has been shown to be an important process in, for instance, extreme marine ecosystems such as hydrothermal vents [7], [8] and in the chemocline of anoxic marine basins [9], [10]. The current consensus is however that chemoautotrophy is a relatively minor process in coastal sediments due to the relatively low growth yields of chemoautotrophic organisms and the competition with chemical oxidation reactions [3]. In addition, true chemoautotrophic bacteria have to compete with mixotrophic and heterotrophic bacteria that are able to oxidize reduced sulfur compounds [11], which could be relevant especially in active coastal sediments receiving large amounts of organic matter. Studies where chemoautotrophy was actually quantified by determining dark carbon fixation rates are rare for typical coastal marine sediments and we have only been able to locate four studies: two on shallow subtidal sediments from the Baltic [12], [13], one study on an intertidal sand flat in the German Wadden Sea [14] and a recent study on three brackish coastal lake sediments in Brazil [15]. However, recent estimates suggest that up to 0.29 Pg C y^−1^ could be potentially fixed by chemoautotrophic microorganisms in near shore and shelf sediments worldwide compared to 0.92 Pg C y^−1^ of mineralization [16], suggesting a major role in the sediment carbon cycle. Finally, the dominant chemoautotrophic bacteria involved in sulfur oxidation are not well known in coastal marine sediments. A recent study identified an uncultered group of Gammaproteobacteria as important players [14], but there may be many other groups involved in the diversity of reoxidation processes that occur in marine sediments.

We studied chemoautotrophy in two intertidal sites with contrasting sulfur chemistry: a site where free sulfide was not detected in the top few centimeters of the sediment and a very active site where high concentrations of free sulfide were found right to the top of the sediment. The main substrates driving chemoautotrophy are therefore expected to be different at both sites, namely free sulfide at the very active site versus iron sulfides in the more typical coastal sediment. Chemoautotrophy rates were determined by incubating sediment cores with stable isotope labeled ^13^C-bicarbonate and measuring labeling in phospholipid derived fatty acids (PLFA). This method both yields estimates of total chemoautotrophy rates and provides an indication of the active bacterial community [17]–[19]. The diversity of Rubisco genes was studied to further indicate possible active chemoautotrophs that use the Calvin cycle for carbon fixation. Finally chemoautotrophy rates were compared with diffusive oxygen fluxes and carbon mineralization rates.

## Materials and Methods

### Description of field sites

Two field sites in the Eastern Scheldt estuary (The Netherlands) were selected, which were expected to show high mineralization rates and have major differences in sulfur chemistry. The site in the Zandkreek area (51°32'41”N, 3°53'22”E) was situated next to a Pacific oyster (*Crassostrea gigas*) bed and was sampled in April 2005 (abbreviation ZK05) and October 2007 (ZK07). The Pacific oyster is an invasive species in the area that was introduced in the Eastern Scheldt around 1970. It stimulates sedimentation and sediment carbon mineralization either by decreasing water currents over the sediment or via pseudo-feces production and biodeposition [20]. Sediments were non-sulfidic in the top 5 centimeters in 2005 and slightly sulfidic below 2 centimeter in 2007 (See Result).

The Rattekaai site (51°26'21”N, 4°10'11”E) was situated at the entrance of a salt marsh creek where macroalgal debris (mainly *Ulv*a derived) accumulates and is buried during winter. The sediment was highly sulfidic right to the top and samples were taken from patches where the sediment was covered with a whitish layer in April 2005 (RK05) and May 2006 (RK06). Based on microscopy, typical *Beggiatoa-*like sulfur-oxidizing bacteria were abundant in the top few millimeters of the Rattekaai sediment, especially in 2005 and to a lesser degree in 2006.

### Ethics statement

The Eastern Scheldt estuary is a Natura 2000 protected area and research permits for both sites were granted by the “Vereeniging Natuurmonumenten” and the Provence of Zeeland.

### Sediment sampling

Undisturbed sediments were sampled with two sizes of polycarbonate core liners. The smaller cores (internal diameter 4.6 cm) contained silicon-filled injection ports at every 0.5 centimeter and were used for measuring chemoautotrophy rates. The larger cores (internal diameter 6 cm) were used for additional measurements of porewater profiles and sediment characteristic, and for measuring mineralization rates. Sediments were sampled at low tide and therefore did not have overlying water. Cores were processed the same day for chemoautotrophy rate measurements and other analyses.

### Chemoautotrophy rates

Chemoautotrophy rate measurements were started by injecting 100 µl of 20 mM NaH^13^CO_3_ (99% ^13^C; Cambridge Isotope Laboratories, Andover, MA, USA) horizontally into the sediment cores at 0.5 cm depth intervals by using the line-injection method [2]. The ^13^C-label was dissolved in artificial seawater lacking calcium or magnesium in order to avoid precipitation [21]. The label was made oxygen free by bubbling with nitrogen gas shortly before injection. Sediment cores were incubated in the dark within 2°C of the in-situ temperature (see Table 1) for various periods of up to 4 days, and were ventilated daily by removing the top stopper for one minute (ZK) or incubated without top stoppers (RK) to circumvent the development of suboxic condition in the headspace. After incubation, sediment cores were sliced to a depth of 5 cm and sediment slices were quickly centrifuged (4500 rpm, 5 min) to collect porewater for concentration and ^13^C analysis of dissolved inorganic carbon (DIC). Sediments were subsequently frozen at −20°C and lyophilized before further analysis. Unlabelled, control cores were also processed.

**Table 1:** Sediment in-situ temperature, sediment characteristics, oxygen consumption rates, carbon mineralization rates, chemoautotrophy rates and yields (averages ± standard deviations, N = 2) for the coastal marine sediments in this study.

Site/Year	Temp. °C	POC^1^ (%)	C/N^1^	O_2_ penetration depth (mm)	O_2_ flux (mmol m^−2^ d^−1^)	C mineralization^2^ (mmol m^−2^ d^−1^)	Chemoautotrophy^2^(mmol C m^−2^ d^−1^)	Yield C/O_2_ (mol C (mol O_2_)^−1^)
RK05	14	-	-	0.45±0.10	17.2±3.0	-	5.5±1.9	0.32±0.11
RK06	17	2.0	10.9	0.23±0.06	192±41	197±36	36.3±4.8	0.19±0.03
ZK05	14	-	-	1.7±0.1	15.0±0.4	-	2.6±0.3	0.17±0.02
ZK07	13	0.6	7.7	0.95±0.06	15.5±1.6	105.9±19.1	2.9±0.2	0.18±0.01

1 Data for 0–1 cm sediment depth. ^2^ Data integrated over 0–5 cm sediment depth.

### PLFA analysis and calculation of chemoautotrophy rates

Lyophilized sediments were analyzed for PLFA concentrations and ^13^C-labeling as described before [22], [23]. In short, PLFA were extracted according to standard protocols and were analyzed by gas chromatography – isotope ratio mass spectrometry (GC-IRMS, Thermo, Bremen, Germany) on an a-polar analytical column (HP5-MS, Agilent, Santa Clara, CA, USA). Stable carbon isotope ratios are reported as δ^13^C ratios on the VPDB scale. Excess ^13^C in individual PLFA was calculated as in Boschker et al [23] and divided by the atom percent excess ^13^C in the DIC pool to calculate actual PLFA synthesis rates. Only very minor labeling was found in poly-unsaturated PLFA typical for Eukarya (see Results) suggesting that PLFA labeling was primarily by Bacteria. We therefore used the labeling data for all common bacterial PLFA in the 12∶0 to 20∶0 range in our calculations and not just the specific bacterial biomarker PLFA [24]. Total bacterial chemoautotrophy rates were determined by summing synthesis rates in all PLFA typically found in bacteria and converted to chemoautotrophic biomass production by dividing by the typical PLFA content of aerobic bacteria (55 mmol PLFA-C (mol biomass C)^−1^ [24], [25]). To study the differences in active chemoautrophic bacterial communities, we performed a principle component analysis (PCA) on log-transformed PLFA ^13^C-labeling data (in Mol%) using the Statistica software package (StatSoft, Tulsa, USA).

### Additional measurements

Oxygen profiles were determined with oxygen microelectrodes (Unisense Ox100, Aarhus, Denmark), which were lowered with a micromanipulator into the sediment until no oxygen was detected. Two profiles were recorded for each duplicate sediment core (four profiles total), which were kept within 2°C of the in-situ temperature. Oxygen fluxes into the sediment were calculated as described in Van Frausum et al. [26] with sediment tortuosity estimated from sediment porosity as in Boudreau and Meysman [27].

Sediment porewater was sampled by slicing duplicate sediment cores in an anaerobic glove-box filled with 3% hydrogen in nitrogen gas (Coy Laboratory Products, Ann Arbor, MI, USA) and slices were centrifuged at 4500 rpm for 10 min at in situ temperature. Samples for sulfide analysis were immediately fixed in zinc acetate and analyzed according to Cline [28]. Samples for ammonium and anion analysis were frozen, and analyzed on a QuAAtro segmented flow analyzer (Seal Analytical, Norderstedt, Germany) and suppressed high performance ion chromatography on a Dionex Ionpac AS-14 column (Thermo, Sunnyvale, CA, USA), respectively. Samples for ^13^C-DIC were added to headspace vials (10 ml) and after acidification analyzed for DIC concentrations and ^13^C-content by elemental analyzer - IRMS [29].

Sediment carbon mineralization rates were determined using the jar method [30]. Sediment cores were sliced as above and were incubated in completely filled centrifuge tubes. Centrifuge tubes containing the sediment were sealed in air-tight incubation bags filled with nitrogen gas to keep them strictly anaerobic and were incubated within 2°C of the in situ temperature for up to 6 days. Sediment porewater was collected and analyzed as described above. Mineralization rates were calculated from DIC and ammonium production with time and ammonium production was converted to carbon mineralization rates by using the sediment C/N ratio (Table 1).

### Rubisco type IA clone libraries

To further study the diversity of chemoautotrophic bacteria that utilize the Calvin cycle for carbon dioxide fixation, Rubisco clone libraries were constructed for both sites in March 2008. Sediments were sampled as described above, and the top 0.5 cm of the cores showing the highest chemoautotrophy rates was collected and immediately frozen at −80°C. Total community DNA was extracted from 0.5 g of wet sediment using the MoBio UltraClean Soil DNA Isolation kit according to protocol (MoBio, Carlsbad, CA, USA).

We developed a new degenerative primer set to specifically amplify Rubisco type IA as this group contains most of the true chemoautotrophic bacteria involved in sulfur and ammonium oxidation [31], [32]. The new primer set also targets *Beggiatoa* like Rubisco sequences [33], which was important as *Beggiatoa*-like bateria were found at the RK site but were not covered by previously published primer sets developed for chemoautotrophic bacteria. The primer set also targets some of the lower branching Type 1B sequences found in unicellular cyanobacteria, and consists of forward primer 571 (GAYTTYACCAARGAYGAYG) and reversed primer 898E (ACRCGGAARTGRATRCC). The primer set was first tested against a positive control (*Thioalkalimicrobium aerophilu* kindly provided by Gerhard Muyzer, Delft Technical University, The Netherlands) and PCR conditions were subsequently optimized to specifically amplify the target sequences from sediment DNA extracts.

The final PCR reaction mixture contained: 2.5 µl 10x standard Taq reaction buffer (without Mg), 3.0 mmol L^−1^ Mg^2+^, 0.2 mmol L^−1^ dNTPS, 0.2 µmol L^−1^ of each primer (571 and 898E), 2 U of NEB Taq DNA polymerase, 5% v/v DMSO, 0.2% w/v BSA and 16 µl autoclaved demi water. The PCR cycling intervals were established as follows: preheating at 94°C for 5 minutes, followed by 40 cycles of denaturation step at 94°C for 1 minute, annealing step at 51°C for 30 seconds and extension at 72°C for 30 seconds. The PCR reaction was finished with a final extension time of 7 minutes at 72°C. PCR products for each sample (RK and ZK) were cloned into *Escherichia coli* Top10 cells using TOPO TA cloning kit (Invitrogen, Carlsbad, CA, USA). Sequencing was performed by a genetic analyzer (Applied Biosystems 3130 Genetic Analyzer, Carlsbad, CA, USA). Editing of the obtained sequences was carried out using the BioEdit software package (http://jwbrown.mbio.ncsu.edu/Bio-Edit/bioedit.html). Primer sequences (T3, T7, 571, 898E) were removed from sequences, then translated to protein sequences, and compared to known sequences using BLAST. Protein sequence alignments and phylogenetic analysis was done in MEGA V [34]. Sequences have been deposited in the GenBank database under accession numbers JQ659214 to JQ659253.

## Results

In spring 2005, both sites (RK05 and ZK05) were studied in an initial test to determine if chemoautotrophy rates could be quantified by ^13^C-DIC labeling of PLFA in the dark. Sites were sampled again in spring 2006 (RK06) and autumn 2007 (ZK07), when a more extensive sampling program was executed.

### Sediment biogeochemistry

**Figure 1:**
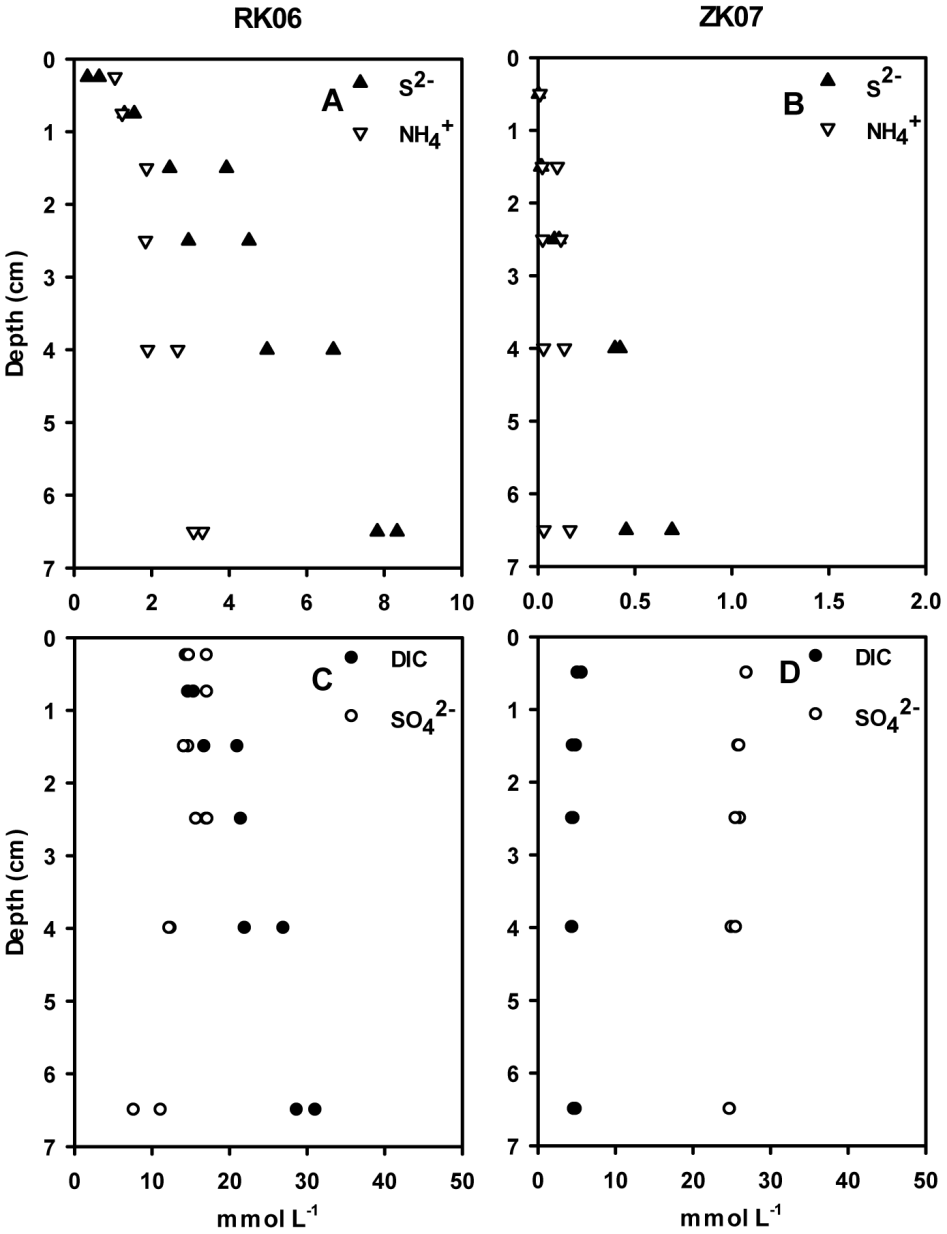
Porewater concentrations for the RK06 and ZK07 sediments. Shown are data for sulfide and ammonium (A, B) and for sulfate and DIC (C, D).

Oxygen penetrated significantly deeper in ZK sediment (1–2 mm) than RK sediment (0.2–0.5 mm, Table 1). At RK06, high concentrations of free sulfide were found in the very top layer of the sediment, whereas sulfide only started to accumulate below 2 cm sediment depth at ZK07 (Fig. 1). Sulfide and ammonium concentrations were more than 10 times higher for RK06 than for ZK07 throughout the sediment column (Fig. 1). In 2005, porewater samples were also taken at both sites and analyzed for sulfide, but samples were taken two weeks before chemoautotrophy measurements and not at exactly the same location, which is especially important for the RK site due to its patchy nature. However, the contrast between the RK and ZK sites was similar with high concentrations of free sulfide at RK05 right to the top of the sediment core and no detectable sulfide in top 5 cm of the ZK05 sediment (results not shown). Porewater concentrations of DIC and SO_4_^2-^ showed little variation with depth for ZK07 strongly indicating bio-irrigation, whereas DIC increased and SO_4_^2-^ decreased with depth for RK06 indicating carbon mineralization by sulfate reduction (Fig. 1).

Diffusive sediment oxygen consumption rates as determined from microelectrode profiles were very high for RK06 with 192 mmol m^−2^ d^−1^ and were approximately 15 mmol m^−2^ d^−1^ for all other samplings (Table 1). The difference between the two RK samplings is probably due to the patchy nature of the site, even though visually similar black sediments with a whitish top layer were sampled in both years. For RK06, anaerobic carbon mineralization rates were about twice as high in the top centimeter (6.8±0.5 µmol C cm^−3^ d^−1^) than in the 1–5 cm layer (3.2±0.8 µmol C cm^−3^ d^−1^), whereas both sediment layers showed similar carbon mineralization rates for ZK07 (0–1 cm, 1.6±0.9 µmol C cm^−3^ d^−1^; 1–5 cm, 2.3±0.3 µmol C cm^−3^ d^−1^). Integrated over the upper 5 cm, anaerobic carbon mineralization rates were 197 and 106 mmol C m^−2^ d^−1^ at RK06 and ZK07, respectively (Table 1). Ammonium production-based mineralization rates agreed with carbon mineralization rates at all sediment depths and ammonium-based mineralization rates in the top layer (0–1 cm) were 8.0±2.0 and 1.1±0.3 µmol C cm^−3^ d^−1^ for RK06 and ZK07, respectively.

### Chemoautotrophy rates

Dynamics of PLFA labeling with ^13^C-bicarbonate were studied in detail for RK06. Substantial labeling could already be detected in the 0–0.5 cm horizon after 4 hours of incubation and, although there was some variation, total PLFA labeling increased linearly with time for up to 4 days (R^2^ = 0.77, n = 8). Similar results were obtained for RK05 and ZK05 as calculated chemoautrophy rates were similar after 2 and 4 days of incubation (data not shown). For RK06, the ^13^C-enrichment in the DIC pool in the 0–0.5 cm of the sediment decreased from 1800±120 ‰ Δδ^13^C (^13^C enrichment of the DIC pool of 1.9%) approximately after 4 hours to 550±110 ‰ Δδ^13^C (0.5% ^13^C) after four days, probably because of exchange with atmospheric carbon dioxide and dilution with DIC produced during organic matter mineralization. Cores from RK06 could not be kept closed at the top because sub-oxic conditions developed within one day due to the very high oxygen consumption rates. The reported chemoautotrophy rates have been corrected for this change in DIC labeling with incubation time.

**Figure 2:**
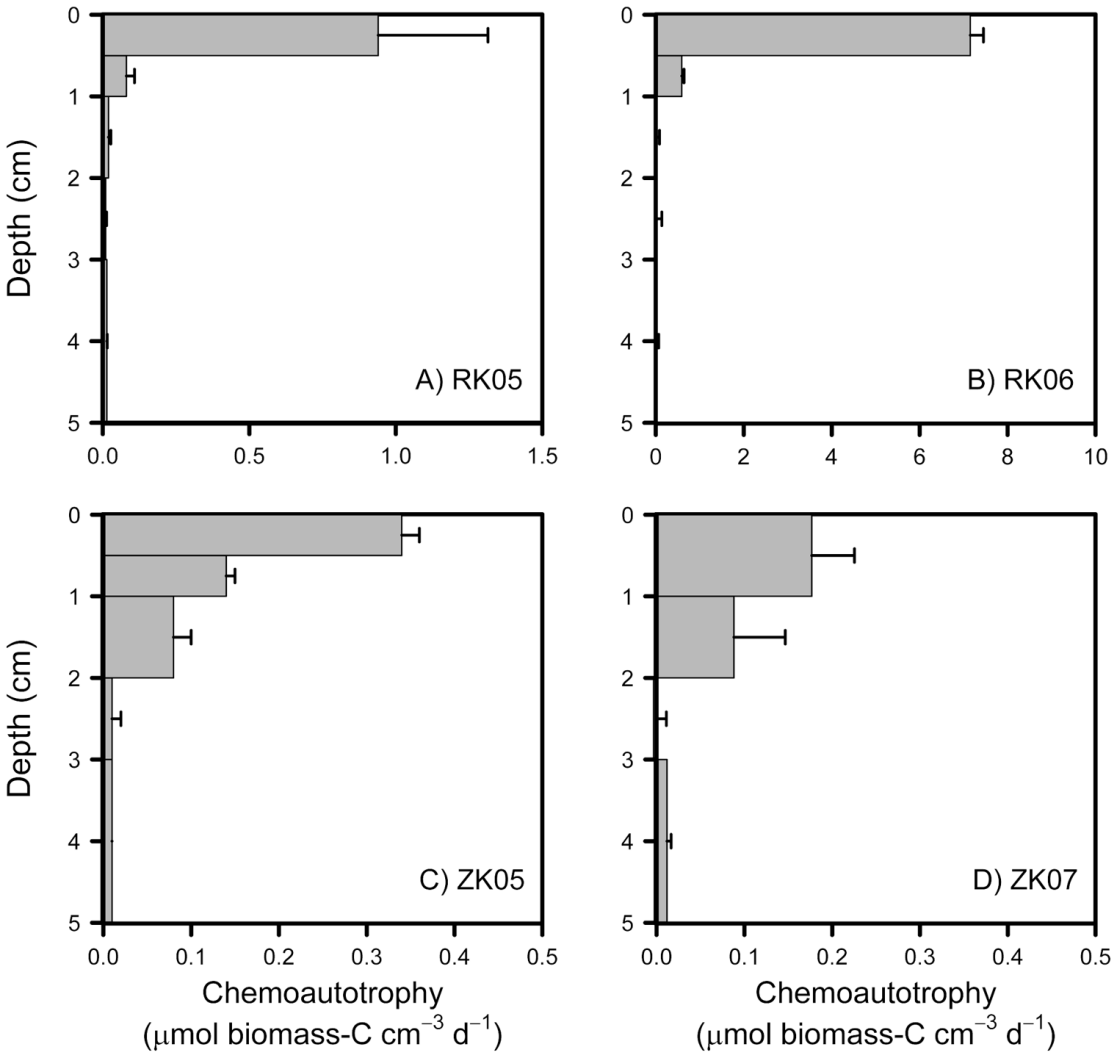
Depth distribution of chemoautotrophy as estimated from ^13^C-DIC PLFA labeling in dark incubations for the RK and ZK sediments.

Chemoautrophy was generally limited to the top centimeter of the sediment especially at the RK site (Fig. 2). For RK06, the main activity (95±1%) was found in the top 0–0.5 cm horizon, which contains the oxic top layer, and below 1 cm depth no activity could be detected. Similar results were obtained for RK05 when depth profiles were determined after 2 and 4 days. Interestingly, chemoautotrophy rates recorded in the top layer of RK06 were similar (7.2 µmol C cm^−3^ d^−1^, Fig 2) to the anaerobic carbon mineralization rates (6.8 µmol C cm^−3^ d^−1^, see above), suggesting balanced CO_2_ production and consumption. For the ZK sediment, highest chemoautotrophy rates were also found in the top layer of the sediment, but activity remained relatively high down to 2 cm depth for both sampling dates.

Depth integrated (0–5 cm), whole-core chemoautotrophy rates ranged from 3–36 mmol C m^−2^ d^−1^ (Table 1). Rates measured at RK06 were very high, about 6 times higher than for RK05, which is probably due to the patchy nature of the site and differences between years (Table 1). Rates for ZK05 and ZK07 were however in the same range and always lower than for the RK site. Whole-core chemoautotrophy rates generally scaled with diffusive oxygen uptake rates and whole sediment chemoautotrophy to oxygen consumption ratios were relatively similar for all sites ranging from 0.17 to 0.32 mol C (mol O_2_)^−1^ (Table 1).

### Active chemoautotrophic bacterial communities

**Figure 3:**
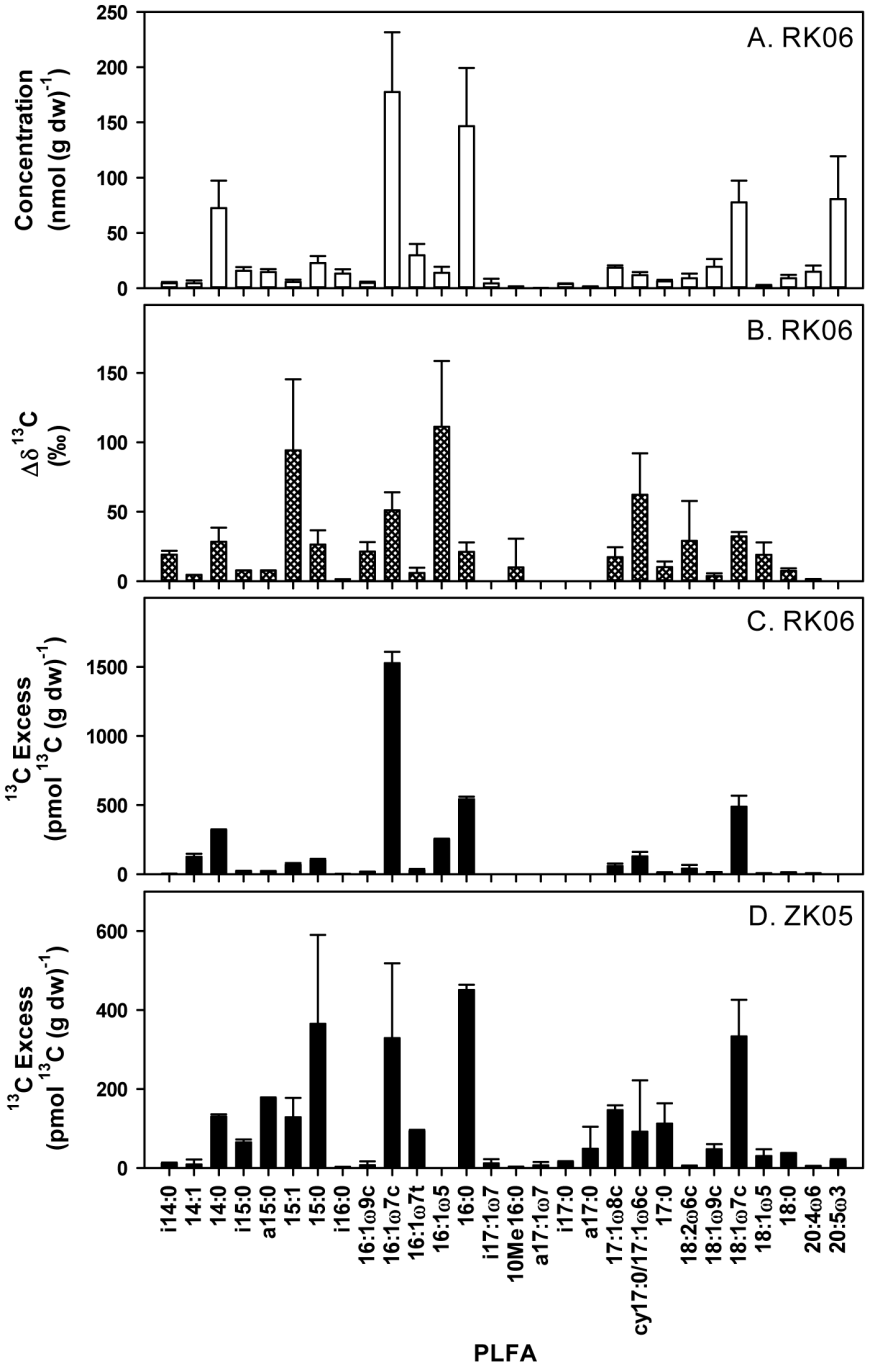
PLFA concentrations (A), Δδ^13^C ratios (B) and excess-^13^C (C) for RK06 (0–0.5 cm horizon) after 1 day of incubation with ^13^C-DIC. The excess-^13^C PLFA data for ZK05 (0-0.5 sediment horizon) after 2 day of incubation are also shown for comparison (D).

As we determined ^13^C-dark fixation rates into biomarker PLFA, the results can also be used to describe and compare active communities of chemoautotrophic bacteria. As an example, Fig. 3 shows the PLFA concentration and labeling data as obtained during this study after one day of incubation for the top layer (0–0.5 cm) of RK06. The PLFA detected were typical for intertidal sediments with high amounts of both bacterial and eukaryote-specific biomarkers; the latter were probably mainly derived from the diatoms growing on the sediment surface as they were dominated by 20∶4ω6 and 20∶5ω3 that occur in high amounts in diatoms (Fig. 3A; [35]). Detection of PLFA labeling is based on the increase in δ^13^C ratios and these were well above detection limits in many PLFA (Fig. 3B; 0.6 to 2‰ Δδ^13^C detection limit depending on compound). The Δδ^13^C ratios were highly variable between PLFA ranging for instance from 0 to 110‰ after one day (Fig. 3B), suggesting that a specific sub-group of the total bacterial community was active. At RK06, PLFA that gained most ^13^C label were 14∶0, 16∶1ω7c, 16∶1ω5, 16∶0 and 18∶1ω7c together explaining 83±4% of the total incorporation into PLFA (Fig. 3C). There were also minor amounts of label recovered in 14∶1, 15∶1, 15∶0, 17∶1ω8 and cy17∶0. Branched, i- and a-PLFA and eukaryote PLFA like 20∶4ω6 and 20∶5ω3 gained very little label even though they were a dominant feature in the PLFA concentration pattern (Fig. 3A & 3C).

The PLFA labeling pattern for the two ZK samplings was very different from the RK site strongly suggesting that different chemoautotrophic communities were active at the two sites. For comparison, the labeling pattern of ZK05 is also presented in Fig. 3D as it showed the largest differences from RK06. Main differences were a much higher labeling in all branched i- and a-PLFA and in several mono-unsaturated PLFA (15∶1, 16∶1ω7t, 17∶1ω8c, 18∶1ω9c and 18∶1ω5) and uneven numbered saturated PLFA (15∶0, cy17∶0 and 17∶0) for both ZK samplings.

**Figure 4:**
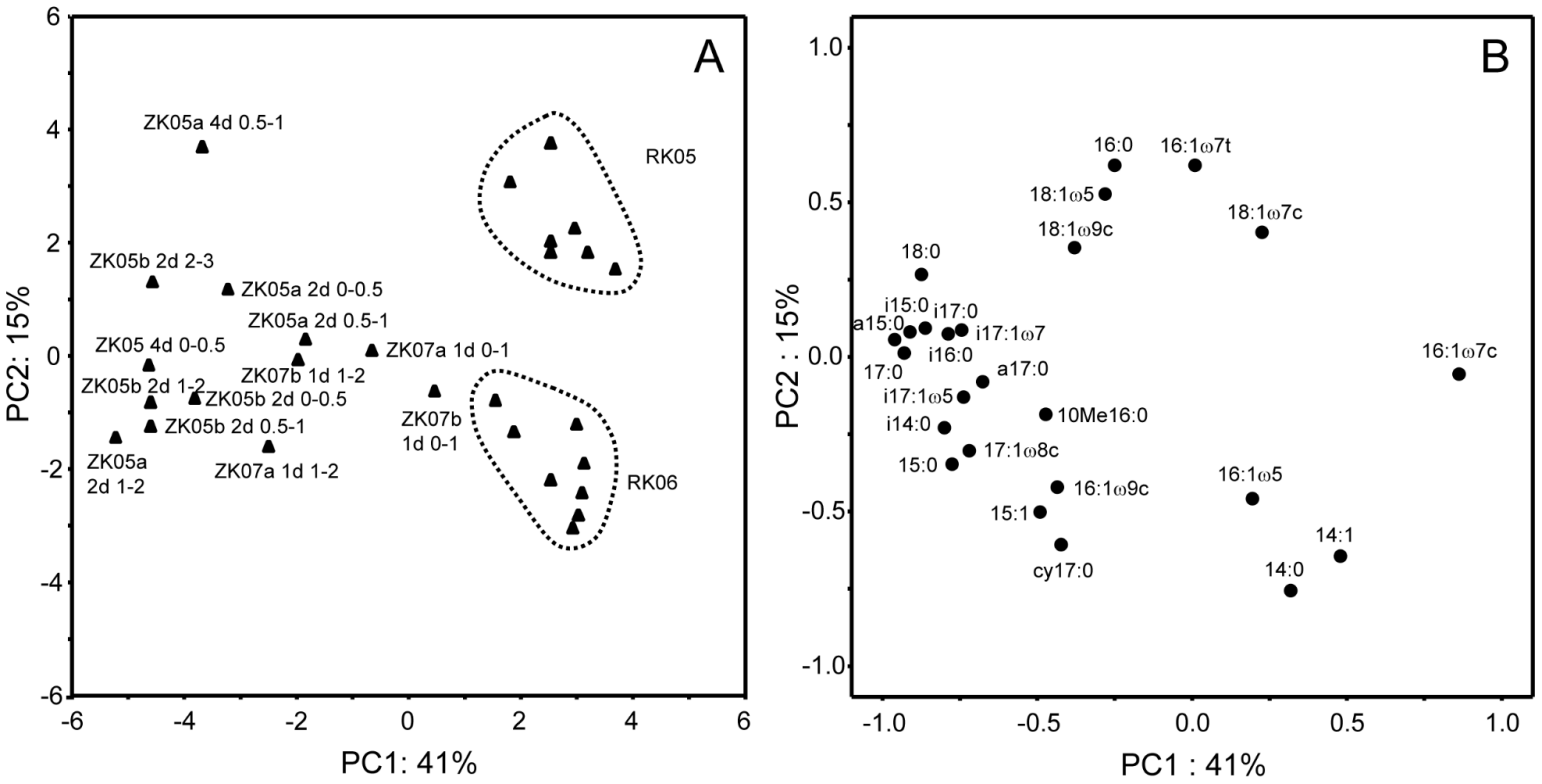
Results of the PCA analysis of the ^13^C-labeling patterns in PLFA for all samples with detectable chemoautotrophy showing the site scores (A) and variable scores (B) for the two PCA axes that explained most of the variance.

To study the differences in PLFA labeling patterns further we performed a PCA analysis for all sediment layers where significant chemoautotrophy was detected (depth ranges RK 0-1 and ZK 0–2 cm; Fig. 4). The first PCA axis explained 42% of the variation found in the data set, whereas the second axis added another 15%. Clustering was mainly based on 16∶1ω7c versus 15∶0, 17∶0, 17∶1ω8c, 18∶0 and all branched PLFA for the first axis similarly as seen in Fig. 3 and 16∶0 and 16∶1ω7t versus 14∶0, 14∶1 and cy17∶0 for the second axis. Clustering of sediment samples was mostly determined by sampling site and sampling year with both RK samplings clustering closely together and more dispersal amongst the samples from the ZK site was observed (Fig. 4A). Some additional variation was found with sediment depth but only for ZK06, where the top layer data (0–1 cm) clustered more closely together with the RK data whereas the deeper layers were shifted towards the ZK05 samples (Fig. 4A). Distribution of label among PLFA did not change substantially with incubation time, suggesting that active chemoautotrophic communities remained similar for up to 4 days. The PCA analysis therefore also showed major difference in active chemoautotrophic bacterial communities between the high free-sulfide RK and low free-sulfide ZK sediments.

There was also indirect evidence of transfer of dark-fixed carbon to fauna: some ^13^C-labelling was detected in bulk sediment PLFA characteristic of Eukarya and therefore fauna, 18∶2w6c for both sites and 20∶4ω6 and 20∶5ω3 for the ZK site (Fig 3C and D).

### Rubisco type IA diversity

To further characterize the chemoautotrophic community, we used a novel primer set to construct clone libraries for the Rubisco Type IA large-subunit gene for both sites in 2008. For the RK site, 17 clones related to Type IA Rubisco were recovered and site ZK yielded 23 clones. We also found a limited number of clones related to unicellular cyanobacteria derived type IB Rubisco especially for the RK site (not shown).

**Figure 5:**
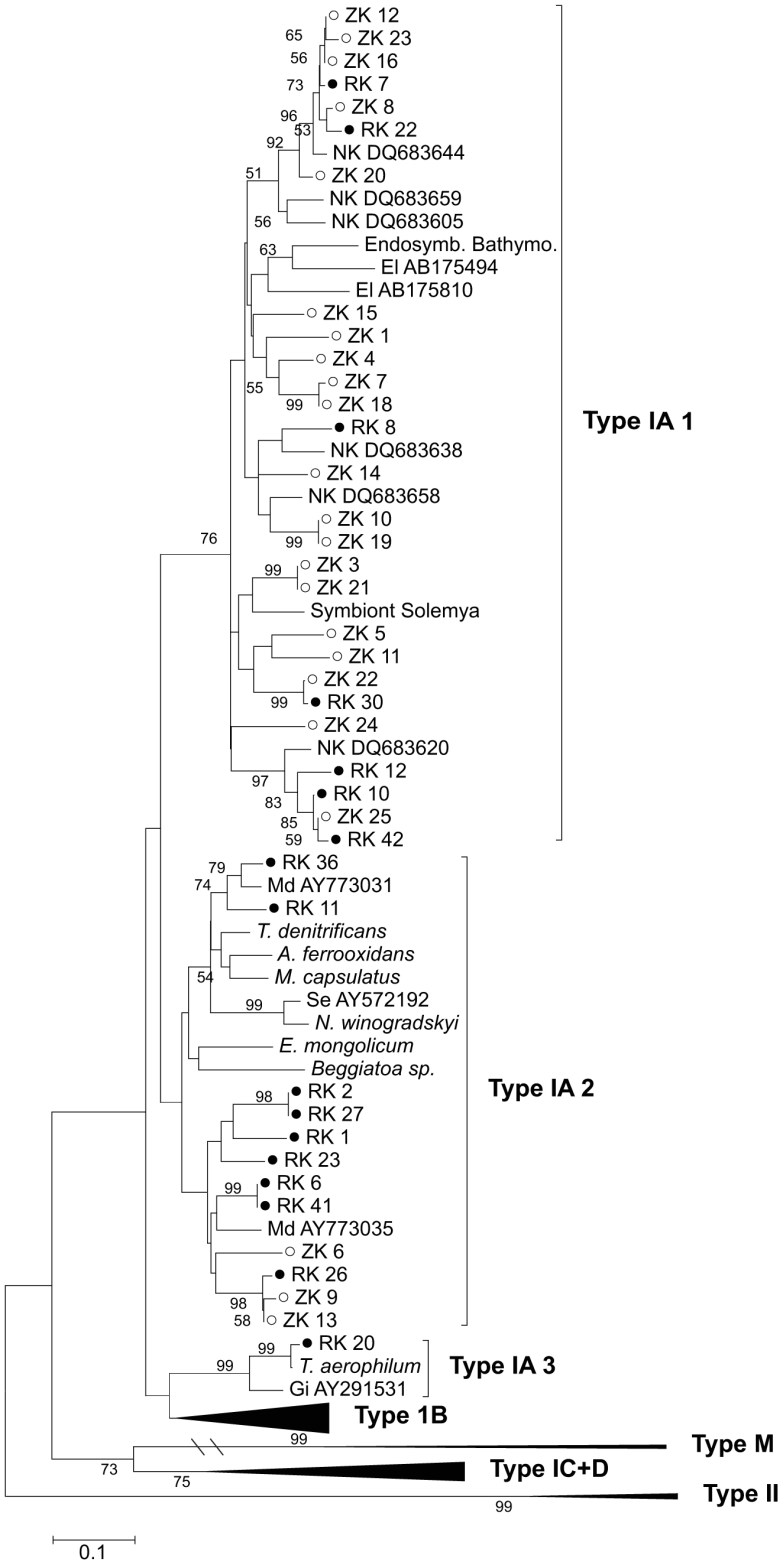
Phylogenetic relationships between Rubisco Type 1A clones recovered from the high free-sulfide RK and low free-sulfide ZK site in 2008 together with relevant sequences of environmental clones from other studies and chemoautotrophic prokaryotes retrieved from Genbank (El: [73]; NK: [32]; Gi: [75]; Md (Madrid et al. unpublished)). The Neighbor-Joining tree is based on amino acid sequence and bootstrap values are based on 1000 times replication.

The phylogenetic relationship for the clones from both sites together with other environmental clones and related micro-organisms is shown in Fig. 5. Type IA clones were found in two main clades labeled type IA 1 and IA 2 (Fig. 5). Rubisco type IA 1 clones were most closely related to uncultured faunal endosymbionts belonging to the Gammaproteobacteria and marine sediment clones from a variety of other studies. Whereas sequences in clade IA 2 were related to various chemoautotrophic sulfur and ammonium oxidizing bacteria and also to other environmental sediment clones. *Beggiatoa* Rubisco type IA also clustered in clade IA 2, but although clones from both RK and ZK were found in this clade they were not closely related to *Beggiatoa* suggesting that they belonged to other groups of chemoautotrophic bacteria. In general rather similar sequences were recovered from both sites, although ZK clones were relatively more abundant in clade IA 1 and RK clones dominated clade IA 2. The results suggest that chemoautotrophic communities, which use the Calvin cycle for carbon fixation, were relatively similar at both sites and that *Beggiatoa* could not be detected at least for the 2008 sampling.

## Discussion

### Rates of chemoautotrophic carbon fixation

We detected very high rates of chemoautotrophic dark fixation in the top layers of two intertidal sediments especially for the RK06 sediment. Volumetric chemoautotrophy rates detected in the top of the RK sediment were comparable to some of the highest sulfate reduction rates detected in marine sediments [36]. These high rates are in part explained because reduced substrates produced by mineralization processes are released throughout the active sediment column whereas the reoxidation by chemoautotrophic bacteria is concentrated in the top of the sediment. Dark fixation rates as measured in our study also includes anapleurotic reactions by heterotrophic bacteria, which may account for up to 5 to 10% of the biomass produced by in all bacteria including heterotrophs [37]–[39]. However, chemoautotrophy rates detected in the top-layer of the RK06 sediment were actually similar to carbon mineralization rates (Fig. 2 and see Results), suggesting that the role of anapleurotic reactions was minimal explaining at most about 5% of the measured dark-fixation rates if one assumes a relatively high heterotrophic growth efficiency of 50% [40]. An additional advantage of measuring dark fixation through ^13^C-labeling of PLFA is that carbon fixed through anaplerotic reactions is not directly utilized in the synthesis of lipids such as fatty acids [39], [41]. Based on oxygen consumption rates and assuming a coupled system, chemoautotrophy explained between 18% (RK06) and 32% (RK05) of the sediment carbon cycling, which would make chemoautotrophy the second or third most important biological carbon cycling process after anaerobic carbon mineralization and possibly photosynthesis by benthic diatoms in these intertidal sediments.

Sulfur oxidizing bacteria are expected to be the main chemoautotrophs in these intertidal sediments; the contribution from ammonium oxidizing bacteria should be less important because about 6 times more sulfide than ammonium is produced during anaerobic carbon mineralization given the typical C:N ratio for marine organic matter [42]. In addition, nitrifying prokaryotes also tend to have lower growth yields per mol of substrate oxidized than sulfur oxidizers [43]. The chemoautotrophy data from our study scaled well with measured diffusive oxygen fluxes with an average whole system yield (± SD) of 0.22±0.07 mol C (mol O_2_)^−1^ (Table 1), which is very similar to the typically reported maximum growth yields for aerobic sulfur oxidizing bacteria of 0.23±0.11 mol C (mol O_2_)^−1^ [44]–[50]. The similarity in C:O_2_ yield between intertidal sediments and sulfur oxidizing bacterial cultures can only be explained if most of the sediment oxygen consumption was indeed used for reoxidation of reduced sulfur and if reoxidation was predominantly performed by obligate sulfur-oxidizing chemoautotrophic bacteria growing close to their maximum reported yields. In addition, chemoautotrophic bacteria should have effectively competed with chemical oxidation processes such as the oxidation of free sulfide with oxygen, as has been shown in gradient systems where oxygen and sulfide are found in close proximity similar to the RK site [3]. Furthermore, even though these coastal sediments receive very high organic matter inputs, our data suggest that the activity by heterotrophic and mixotrophic sulfur-oxidizing bacteria was also limited as this should have led to lower C:O_2_ yields. Competition between autotrophic and heterotrophic or mixotrophic sulfur oxidizing bacteria depends to a large extent on the sulfur/organic substrate ratio with low ratios supporting heterotrophic sulfur oxidation [11], [51], but this apparently didn't play a role in the studied sediments possibly due to strong competition for organic substrates by other heterotrophic bacteria. Our results suggest that dark fixation rates as determined by ^13^C-bicarbonate labeling of PLFA yield realistic chemoautotrophy rates in relation to sediment biogeochemistry and that chemoautotrophic bacteria were growing with high efficiency independent of sediment sulfur chemistry.

Chemoautotrophy rate data are available from only four other studies on coastal marine sediments, all of which measured total dark fixation rates by determining ^14^C-DIC incorporation into POC [12]–[15]. It should be noted that two of these studies are based on laboratory incubations with homogenized sediments [12], [15]. Thomsen and Kristensen [12] reported maximum rates of approximately 0.35 µmol C cm^−3^ d^−1^ (averaged over the same depth as in the present study) similar to site ZK, whereas rates found by Enoksson and Samuelsson [13] and Lenk et al [14] are lower at about 0.12 µmol C cm^−3^ d^−1^. The data collected by Thomsen and Kristensen [12] yield a C:O_2_ ratio of 0.24 mol C (mol O_2_)^−1^ similar to our data, whereas the chemoautrophy rates reported by Enoksson and Samuelsson [13] are substantially higher than expected from their oxygen consumption rates (C:O_2_ ratio about 1.2 mol C (mol O_2_)^−1^). The C:O_2_ ratios from Enoksson and Samuelsson [13] can not be readily explained in relation to the reported growth yields of sulfur reducing bacteria [44]–[50], suggesting that the chemoautotrophy rates for this sediment may have been substantially overestimated possibly due to the incomplete removal of ^14^C-DIC label. Santoro et al [15] found much lower yields of about 0.025 mol C (mol O_2_)^−1^ for three brackish coastal lake sediments. Lenk et al [14] also reported ^14^C-POC based chemoautotrophy rates of 4.16±0.03 mmol C m^−2^ d^−1^ for an intertidal flat consisting of permeable sands, which related well to sediment sulfide fluxes [14], [52]. Lenk et al [14] did not report oxygen consumption rates. However, potential oxygen fluxes of approximately 70 mmol O_2_ m^−2^ d^−1^ have been reported for the same sand flat [53], suggesting a relatively low C:O_2_ yield of about 0.06 mol C (mol O_2_)^−1^. Oxygen fluxes may have been overestimated because they are potential rates based on aerobic incubations with sediments from different horizons that may not always be oxic. The depth distributions of chemoautotrophy of Enoksson and Samuelsson [13] and Lenk et al. [14] were different from our study as they both found substantial rates deeper in the sediment up to a depth of 10 cm. Lenk et al [14] studied a permeable sediment where oxidants such as oxygen are transported deep into the sediment by advective porewater flows [53], which may explain the high chemoautotrophy rates deeper in the sediment. The chemoautotrophy rates in our study at the RK06 (3.2 to 6.8 µmol C cm^−3^ d^−1^) are the highest reported so far for coastal marine sediments.

Furthermore, our results suggest that chemoautotrophically fixed carbon may also be an important food source in the microbial food web in these typical coastal sediments. Chemoautotrophy rates were similar to carbon mineralization rates in the top layer of the RK06 sediment (Fig. 2 and Results). Net consumption of DIC related to the chemoautotrophy has been indicated in the top layer of active coastal sediments [12], [54]. This suggests that the production by chemoautotrophs dominated the microbial food web and that heterotrophic bacterial secondary production was less important (if one assumes a growth efficiency of 50% for heterotrophic bacteria). Santoro et al [15] measured growth of both chemoautotrophic and heterotrophic bacteria in three brackish lakes and found that chemoautotrophy could explain up to 50% of the heterotrophic bacterial growth. Additionally, chemosynthetically produced biomass may potentially be an important source of energy fueling the benthic food web. We indeed detected limited labeling in eukaryotic fauna-derived PLFA for both sediments (Fig 3C & D), which suggests that sediment fauna may in part be feeding on chemoautotrophic bacteria. Chemoautotrophic bacteria support the food web in many extreme marine ecosystems like hydrothermal vents and mud volcanoes with limited organic matter inputs [8], [55]. Based on our results, the role of chemoautotrophic carbon fixation in the benthic food web of coastal marine sediments should receive more attention.

### Communities of chemoautotrophic bacteria

We detected clear difference in PLFA labeling patterns for the ZK and RK sediments suggesting that the active chemoautotrophic bacterial communities were substantially different (Fig. 3 and 4). The classical sulfur and ammonium oxidizing bacteria predominantly contain even-numbered saturated and mono-unsaturated PLFA such as 14∶0, 16∶1ω7c, 16∶0 and 18∶1ω7c [17], [55]–[60]. For the RK sediment, most of the label was indeed recovered in these typical PLFA and the PLFA labeling pattern for instance closely resembles the PLFA composition of *Beggiatoa* especially for RK05 (Fig. 3 and [55], [56]). Some types of sulfur oxidizing thiobacilli also contain uneven numbered PLFA like cy17∶0 [17], [57], and label was indeed recovered in these PLFA especially at both ZK samplings and at RK06 suggesting that these groups may also be active in coastal sediments. In view of the high free sulfide and ammonium concentrations in the porewater, the PLFA labeling at the RK site was therefore in agreement with the activity of typical chemoautotrophic sulfur and ammonium oxidizing bacteria.

In contrast, major amounts of label were also found in branched i- and a-PLFA at the ZK site, which have not been reported in the classical chemoautotrophic sulfur oxidizing and nitrifying bacteria. However, several groups of sulfate reducing bacteria contain large amounts of branched PLFA [61]–[63]. In addition, significant amounts of label were recovered in i17∶1ω7, a17∶1ω7, 17∶1ω8c and cy17∶0/17∶1ω6c that have been suggested as specific biomarkers for certain groups of sulfate reducing bacteria [61]–[63], suggesting that sulfate reducing bacteria may be important chemoautotrophs in the ZK sediment. Chemoautotrophic growth can be found in two groups of sulfate reducing bacteria namely those that use hydrogen gas as substrate [64] and sulfur disproportionating bacteria that gain energy from inorganic fermentation of substrates such as thiosulfate and elemental sulfur to sulfate and sulfide [65], [66]. Both hydrogen turnover and disproportionation reactions have been shown to be important processes in anaerobic marine sediments [67], [68] and they have been indicated to possibly support chemoautotrophy in coastal sediments [12]. For the ZK07 sediment, the labeling in these branched PLFA became progressively more important with depth (Fig. 4), suggesting that it may indeed be associated with these anaerobic processes. Branched PLFA have also been found in anammox bacteria [69], but these also contain 10Me16∶0 which showed no or limited labeling in our study (Fig. 3) suggesting that they were not important as chemoautotrophs. This is in agreement with studies that found that denitrification and not anammox is the dominant nitrate consuming process in active coastal sediments [70], [71]. The high labeling in branched PLFA as detected at the low free-sulfide ZK site therefore indicates that anaerobic chemoautotrophy most likely by bacteria related to sulfate reducers may be important in coastal sediments.

We also studied the diversity of chemoautotrophic bacteria that use the Calvin cycle for carbon fixation by constructing clone libraries of the Rubisco type IA gene. Clones detected fell in two main clades: one related to uncultured endosymbiontic sulfur-oxidizing Gammaproteobacteria (clade IA 1) and another related to free living chemoautotrophic sulfur and ammonium oxidizing bacteria (clade IA 2, Fig. 5). Although clade IA 1 sequences were most closely related to endosymbionts, they are also commonly found in related uncultured free-living bacteria [14], [72]. These two Rubisco clades are usually also detected in other environmental studies on marine sediments [32], [73]. Lenk et al [14] also showed that that free-living Gammaproteobacteria related to sulfur-oxidizing endosymbionts were important for dark carbon fixation in a permeable tidal flat sediment. The differences in Rubisco clone distribution between the two sediments are most likely due to differences in the availability of reduced sulfur compounds namely free sulfide at the RK site and iron sulfides at the ZK site. However, it seems unlikely that these differences in clone distribution explain the contrasting PLFA labeling patterns from the two sites. First, the differences in Rubisco clone distribution appear to be less site-dependent compared to the PLFA labeling patterns that showed major differences between sites. Second, the detected clones are related to bacteria that typically do not contain i- and a-branched PLFA as found for the chemoautotrophs from the ZK site. As discussed before, the PLFA composition of both the typical chemoautotrophic sulfur and ammonium oxidizers in clade IA 2 and Gammaproteobacteria like the ones detected in clade IA 1 is usually dominated by straight saturated and un-saturated compounds similar to the labeling patterns at the RK site [17], [57], [58]. Third, sulfate reducing bacteria typically fix inorganic carbon by either the reversed TCA-cycle or the reductive acetyl-CoA pathway [64] and are therefore not targeted in our Rubisco assay. The dominance of Gammaproteobacteria among the Rubisco clones is therefore also agreement with the PLFA labeling patterns especially for RK and to lesser degree for ZK.

To conclude, we detected very high rates of chemoautotrophy in two active intertidal sediments and our results indicate that chemoautotrophic carbon fixation is an important part of the carbon cycle of coastal sediments. Clear differences were found in active chemoautotrophic bacterial communities probably caused by differences in reduced sulfur compounds available in the two sediments. Our study suggests that anaerobic bacteria related to sulfate reducers played an important role at the low-sulfide ZK site while typical sulfur-oxidizers, probably Gammaproteobacteria, were more important at the free-sulfide RK site. Chemoautotrophic Archaea such as the ammonium-oxidizing Crenarcheoata widely found in marine sediments [74] were not considered in the present study, but their activity could be studied by determining ^13^C labeling in Archaeal ether-lipids [41]. The number of coastal sediments where chemoautotrophic carbon fixation rates have been determined is still very low especially compared to studies on other aspects of the sedimentary carbon cycle. Besides our study we are aware of only four other studies where chemoautotrophy rates were quantified in coastal marine and brackish lake sediments [12]–[15]. Hence, chemoautotrophy in coastal marine sediments as driven by reoxidation processes should receive more attention in future studies given its importance in the carbon cycle of coastal sediments and the potential role in the benthic food web.
